# OryzaGenome2.1: Database of Diverse Genotypes in Wild *Oryza* Species

**DOI:** 10.1186/s12284-021-00468-x

**Published:** 2021-03-04

**Authors:** Hiromi Kajiya-Kanegae, Hajime Ohyanagi, Toshinobu Ebata, Yasuhiro Tanizawa, Akio Onogi, Yuji Sawada, Masami Yokota Hirai, Zi-Xuan Wang, Bin Han, Atsushi Toyoda, Asao Fujiyama, Hiroyoshi Iwata, Katsutoshi Tsuda, Toshiya Suzuki, Misuzu Nosaka-Takahashi, Ken-ichi Nonomura, Yasukazu Nakamura, Shoko Kawamoto, Nori Kurata, Yutaka Sato

**Affiliations:** 1 Department of Agricultural and Environmental Biology, Graduate School of Agricultural and Life Science, The University of Tokyo, Bunkyo 1-1-1, Tokyo, 113-8657 Japan; 2 King Abdullah University of Science and Technology, Computational Bioscience Research Center, Biological and Environmental Sciences & Engineering Division, Thuwal, 23955-6900 Saudi Arabia; 3 Dynacom Co., Ltd., World Business Garden, Marive East 25F, 2-6-1, Nakase, Mihama-ku, Chiba-shi, Chiba, 261-7125 Japan; 4 National Institute of Genetics, Yata 1111, Mishima, Shizuoka 411-8540 Japan; 5 Institute of Crop Science, NARO, Kannondai 2-1-2, Tsukuba, Ibaraki 305-8518 Japan; 6 RIKEN Center for Sustainable Resource Science, 1-7-22 Suehiro-cho, Tsurumi-ku, Yokohama, Kanagawa 230-0045 Japan; 7 National Center for Gene Research, Institute of Plant Physiology and Ecology, Shanghai Institutes for Biological Sciences, Chinese Academy of Sciences, 500 Caobao Road, Shanghai, China

**Keywords:** Database, Genome diversity, *Oryza*, NIG wild *Oryza* collection, Oryzabase, *Oryza rufipogon*, Polyploidy

## Abstract

**Background:**

OryzaGenome (http://viewer.shigen.info/oryzagenome21detail/index.xhtml), a feature within Oryzabase (https://shigen.nig.ac.jp/rice/oryzabase/), is a genomic database for wild *Oryza* species that provides comparative and evolutionary genomics approaches for the rice research community.

**Results:**

Here we release OryzaGenome2.1, the first major update of OryzaGenome. The main feature in this version is the inclusion of newly sequenced genotypes and their meta-information, giving a total of 217 accessions of 19 wild *Oryza* species (*O. rufipogon*, *O. barthii*, *O. longistaminata*, *O. meridionalis*, *O. glumaepatula*, *O. punctata*, *O. minuta*, *O. officinalis*, *O. rhizomatis*, *O. eichingeri*, *O. latifolia*, *O. alta*, *O. grandiglumis*, *O. australiensis*, *O. brachyantha, O. granulata*, *O. meyeriana*, *O. ridleyi*, and *O. longiglumis*). These 19 wild species belong to 9 genome types (AA, BB, CC, BBCC, CCDD, EE, FF, GG, and HHJJ), representing wide genomic diversity in the genus. Using the genotype information, we analyzed the genome diversity of *Oryza* species. Other features of OryzaGenome facilitate the use of information on single nucleotide polymorphisms (SNPs) between *O. sativa* and its wild progenitor *O. rufipogon* in rice research, including breeding as well as basic science. For example, we provide Variant Call Format (VCF) files for genome-wide SNPs of 33 *O. rufipogon* accessions against the *O. sativa* reference genome, IRGSP1.0. In addition, we provide a new SNP Effect Table function, allowing users to identify SNPs or small insertion/deletion polymorphisms in the 33 *O. rufipogon* accessions and to search for the effect of these polymorphisms on protein function if they reside in the coding region (e.g., are missense or nonsense mutations). Furthermore, the SNP Viewer for 446 *O. rufipogon* accessions was updated by implementing new tracks for possible selective sweep regions and highly mutated regions that were potentially exposed to selective pressures during the process of domestication.

**Conclusion:**

OryzaGenome2.1 focuses on comparative genomic analysis of diverse wild *Oryza* accessions collected around the world and on the development of resources to speed up the identification of critical trait-related genes, especially from *O. rufipogon*. It aims to promote the use of genotype information from wild accessions in rice breeding and potential future crop improvements. Diverse genotypes will be a key resource for evolutionary studies in *Oryza*, including polyploid biology.

**Supplementary Information:**

The online version contains supplementary material available at 10.1186/s12284-021-00468-x.

## Background

Rice is an essential food for humankind, playing a critical role in food security. In addition, rice is a well-established model monocot in plant science, enabling advanced genomic breeding approaches for rapid development of improved cultivars. One such approach is genomic introgression from either closely or distantly related wild *Oryza* species into cultivated rice species, with the aim of exploiting agronomically advantageous traits from diverse wild relatives.

While a massive number of Asian cultivated rice (*Oryza sativa* L.) genotypes have been made available to the public (Alexandrov et al. 2015) (Wang et al. 2018), few wild *Oryza* genotypes have been released. To provide an open-access platform for genomic information on highly diverse wild *Oryza* species, OryzaGenome (Ohyanagi et al. 2016) was launched in 2015. The original version included the basic function of SNP viewer for 446 *O. rufipogon*, in addition to genotype information of 446 *O. rufipogon* accessions and 17 *Oryza* accessions. It also included hyperlinks to phenotypic and habitat information in Oryzabase (Kurata and Yamazaki 2006) to provide a functional genomics approach for the wild *Oryza* research community.

Here, we release OryzaGenome2.1 (http://viewer.shigen.info/oryzagenome21detail/index.xhtml), a major update. This release features newly sequenced genotypes and their meta-information, for a total of 217 wild *Oryza* accessions from 19 *Oryza* species (Table 1). It also provides an intuitive single nucleotide polymorphism (SNP) Effect Table functionality using 33 deep-sequenced *O. rufipogon* accessions in addition to a SNP Viewer with new information tracks for 446 imputed *O. rufipogon* genotypes. Because the 19 wild species belong to 9 genome types, this release will provide access to the genomic diversity within the genus *Oryza* for comparative and evolutionary genomics, as well as the potential for further improvement in rice breeding practices.

**Table 1:** List of information released in OyrzaGenome2.1

	217 Wild *Oryza* Genome Sequences	33 Deep-sequenced *O. rufipogon*	Imputed 446 *O. rufipogon* Genotypes
Number of Accessions	217^a^	33	446
Species	19 wild *Oryza* species	*O. rufipogon*	*O. rufipogon*
Raw NGS data	✓	✓	✓
Imputation-free variants		✓	
SnpEff-based Variant Lists		✓	
Imputed variants			✓

^a^Including 33 Deep-sequenced *O. rufipogon*

## Materials and Methods

### Reference Genome Information

We used Os-Nipponbare-Reference-IRGSP-1.0 (*O. sativa* ssp. *japonica* ‘Nipponbare’) as a reference genome sequence (Kawahara et al. 2013). For gene annotations, we used MSU Rice annotations (MSU Rice Genome Annotation Project Team 2015) and RAP-DB annotations (Sakai et al. 2013).

### Genomic Data for 217 *Oryza* Genotypes

Of the 217 wild *Oryza* accessions (Table 1, Supp. Table 1), 213 are maintained at the National Institute of Genetics (Nonomura et al. 2010), and the remaining 4 were obtained from IRRI. DNA was extracted from leaves and sequenced as described (Shenton et al. 2020). Short-read Illumina sequencing was performed on an Illumina HiSeq sequencer with paired reads (Illumina, San Diego, CA). Each raw sequence is available via the National Center for Biotechnology Information (NCBI) and DNA Data Bank of Japan (DDBJ) Sequence Read Archive (DRA) system.

### *K*-Mer Analysis

*K*-mer analysis was conducted using GenomeScope (v1.0.0) following the instruction of the software (Vurture et al. 2017). *K*-mer frequency was counted using Jellyfish ver. 2.2.6 with the *k*-mer size of 21 and the “cannonical kmers” option (−C) (Marcais and Kingsford 2011). The resultant *k*-mer histogram was subjected to GenomeScope to estimate genomic properties such as genome size, repeat content, and heterozygosity. For several accessions, the model fit of GenomeScope did not converge, possibly because of the low sequencing coverage.

### Genomic Data and VCF Files for 33 *O. rufipogon* Accessions

Thirty-three *O. rufipogon* accessions (Table 1, Supp. Table 1) were genotyped at higher coverage (average 19.5×). To process the resequencing reads from these accessions, we modified the analytical workflow originally developed for the TASUKE+ variant browser of the Rice Annotation Project Database (RAP-DB; https://rapdb.dna.affrc.go.jp/genome-wide_variations/Analysis_workflow_for_detection_of_genome-wide_var.html) (Kumagai et al. 2019). Briefly, paired-end reads preprocessed by Trimmomatic v. 0.38 (Bolger et al. 2014) were mapped on the IRGSP1.0 reference genome (including organellar and unanchored contig sequences) using the bwa-0.7.17 mem (Li and Durbin 2010) algorithm with the default options. PCR duplicates were removed using MarkDuplicates of the Picard package v. 2.18.17 (http://broadinstitute.github.io/picard). Variants were called by HaplotypeCaller of the Genome Analysis Toolkit (GATK, v. 4.0.11.0) (McKenna et al. 2010). They were filtered using VariantFiltration of GATK with filter expressions of “DP < 2 || QD < 2.0 || FS > 60.0 || MQ < 40.0 || SOR > 3.0 || MQRankSum < −12.5 || ReadPosRankSum < −8.0” for homozygous variants. Finally, the variants were annotated using SNPeffect v. 4.3 (Cingolani et al. 2012) based on the gene annotation available at RAP-DB (release 26 Nov 2018).

**Fig. 1:**
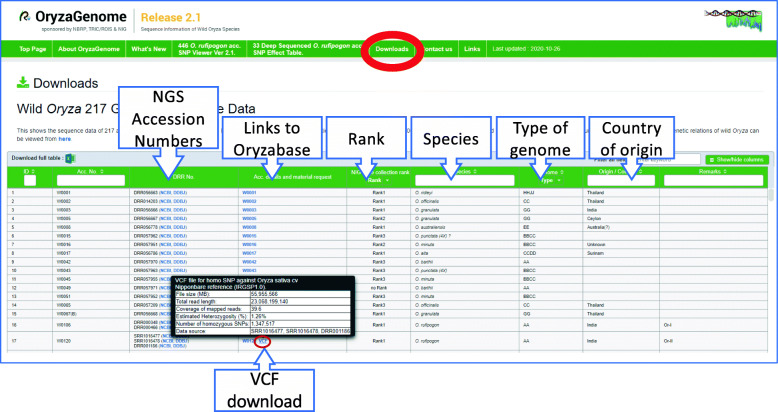
Downloads page. Information on 217 wild *Oryza* genotypes can be searched by using the conditions in multiple columns. Information on the 33 Deep-sequenced *O. rufipogon* Accessions can be also obtained from this page

The raw sequence data as well as variant information in Variant Call Format (VCF) files are accessible from the Downloads page (Fig. 1). The analytical pipeline implemented in Common Workflow Language is available from https://github.com/nigyta/rice_reseq.

### Genomic Data for 446 Imputed *O. rufipogon* Variants

Imputed genome data were obtained as follows. Whole-genome information in OryzaGenome v. 1 Variant List (Ohyanagi et al. 2016) (http://viewer.shigen.info/oryzagenome/mapview/​VariantView.do?action=Downloads) was downloaded and re-imputed by beagle 4.0 software (Browning and Browning 2007) (https://faculty.washington.edu/browning/beagle/b4_0.html). This file contains phased, non-missing genotypes for all non-reference positions and is available from the Downloads page.

### System Architecture and Software

OryzaGenome2.1 is implemented on a UNIX server with CentOS v. 7, with Apache/Tomcat and Apache/GlassFish web servers and a PostgreSQL Database server.

Java and C++ were used as server-side application languages. JavaScript was used to implement client-side rich applications. The JavaScript libraries jQuery (http://jquery.com), DataTables (https://www.datatables.net/), Magnific Popup (http://dimsemenov.com/plugins/​magnific-popup/), Google chart tools (https://developers.google.com/chart/), Prototype (http://prototypejs.org/), and script.aculo.us (https://script.aculo.us/) were used. Other conventional utilities for UNIX computing were installed on the server as necessary. All the OryzaGenome2.1 resources are stored on the server and are available at http://viewer.shigen.info/oryzagenome21detail/index.xhtml.

## Results and Discussion

### Genotypes and Batch Downloads

OryzaGenome release 2.1 provides the genotypes of 217 highly diverse accessions from 19 wild *Oryza* species (average coverage 18×) (Table 1; Supp. Table 1). Among the 217 *Oryza* accessions, 33 *O. rufipogon* accessions were genotyped at higher coverage (average 19.5×), and their variants against the *japonica* reference genome (Kawahara et al. 2013) are available in VCF files (Table 1; Supp. Table 1). OryzaGenome2.1 provides wild *Oryza* genotype information in five categories: (1) 217 *Oryza* Genotypes; (2) 33 *O. rufipogon* Genotypes and Variants; (3) SNP Viewer for 446 Imputed *O. rufipogon* Genotypess, with new information tracks; (4) SNP Effect Table function to search for the effects of variants in 33 *O. rufipogon* accessions against IRGSP1.0; and (5) Misc Downloads.

### 217 *Oryza* Genotypes

In the Downloads section of OryzaGenome2.1, next-generation sequencing (NGS)-derived genotypes of 217 accessions from 19 wild *Oryza* species (*O. rufipogon*, *O. barthii*, *O. longistaminata*, *O. meridionalis*, *O. glumaepatula*, *O. punctata*, *O. minuta*, *O. officinalis*, *O. rhizomatis*, *O. eichingeri*, *O. latifolia*, *O. alta*, *O. grandiglumis*, *O. australiensis*, *O. brachyantha, O. granulata*, *O. meyeriana*, *O. ridleyi*, and *O. longiglumis*) are provided. The 19 wild species belong to 9 genome types (AA, BB, CC, BBCC, CCDD, EE, FF, GG, and HHJJ), representing much more genomic divergence than in the previous version. Each accession has been genotyped by Illumina paired-end short-read DNA sequencing (100- to 150-bp read length for one end) of over 8× coverage (average 18× coverage). Users can browse the sequencing platform, total bases, coverage, and file size in a pop-up window by mouse-over action at the accession numbers that are hyperlinked to NCBI and DDBJ on the Downloads page (Fig. 1). The meta-information for these accessions is also available on the Downloads page. Data can be selected and sorted by changing search conditions such as species, genome type, growth habit (annual or perennial), rank in the core collection, and origin (Fig. 1). To obtain phenotype information on each accession, links are provided to web pages in Oryzabase (Kurata and Yamazaki 2006) describing the characteristics of each accession (Fig. 1), as well as to request resources.

Using the genotype information in Oryzagenome2.1, we analyzed the genome diversity of genus *Oryza*. *K*-mer analysis was conducted to estimate the size of genome, the content of repetitive sequences and the heterozygosity in the genome of each accession (Supplementary Table 2). It turned out that there is a significant degree of variance in the genome size ranging approximately 250 ~ 1000 Mbp and this is largely concomitant with previous reports (Stein et al., 2018). The size of the genome tends to be similar among the same genome type and among the same species. The genome size of *O. brachyantha* and *O. ridleyi* are the smallest and the largest, respectively (Supplementary Fig. 1A). There is a positive linear correlation between genome size and repeat length among *Oryza* including diploid and tetraploid species (Supplementary Fig. 1A). However, the content of repetitive sequences in tetraploid species is rather constant, despite the large difference in the genome size (Supplementary Fig. 1B). This suggests that the genome size of ancestral diploid species may have the major impact on that of tetraploid species (Shenton et al., 2020). Overall, genotype data available from Oryzagenome2.1 would contribute to analyze various aspect of genome diversity seen among genus *Oryza*.

### 33 *O. rufipogon* Genotypes and Variants

Because *O. rufipogon* is the progenitor of Asian cultivated rice, *O. sativa*, we paid special attention to it and obtained higher coverage (average 19.5×) for 33 accessions of *O. rufipogon*. The raw sequence data of these accessions, as well as VCF files containing information on variants against Os-Nipponbare-Reference-IRGSP-1.0, are provided on the Downloads page (Fig. 1). Users can browse the coverage, estimated heterozygosity, number of homozygous polymorphisms, and file size in a pop-up window by mouse-over action at the VCF download icons. In addition, the numbers of homozygous SNPs, and small insertions and deletions in 33 *O. rufipogon* accessions are provided in Supplementary Table 3.

### SNP Viewer with New Information Tracks for 446 Imputed *O. rufipogon* Genotypes

**Fig. 2:**
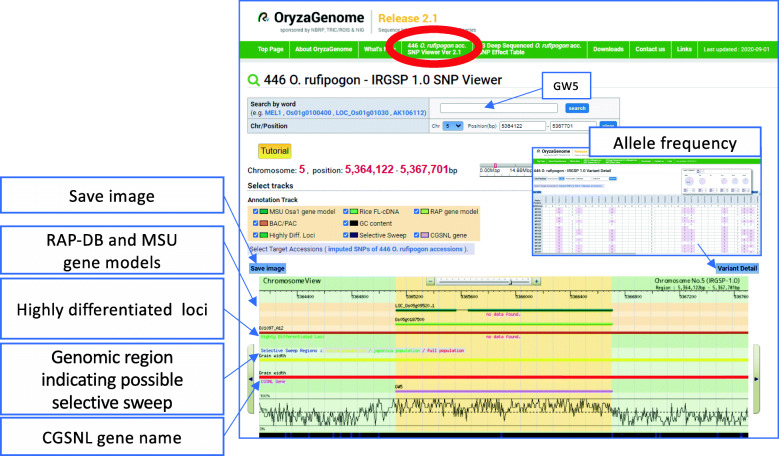
Genome-browser-oriented SNP Viewer for the 446 imputed *O. rufipogon* variants showing the Os05g0187500 gene region. A new function, based on previous data (Huang et al. 2012), graphically represents genomic regions in *O. sativa* where selective sweeps may have occurred: red bars, whole population; yellow bars, only in *indica* populations; green bars, only in *japonica* populations. This release also graphically shows regions with higher mutation rates (i.e., highly differentiated loci). Allele frequencies in a region can be displayed by clicking on the “Variant Detail” link

The genome-browser-oriented SNP Viewer (Fig. 2) for the 446 imputed *O. rufipogon* genotypes (see the Misc Downloads section) is updated in this release. Chromosome coordinates, genome sequences, and gene annotations are based on the latest *japonica* reference genome, Os-Nipponbare-Reference-IRGSP-1.0 (Kawahara et al. 2013). In addition, this version graphically presents genomic regions where selective sweeps may have occurred during domestication of *O. sativa* from *O. rufipogon* populations (Fig. 2) according to data published in Huang et al. (2012). Genomic regions in *O. sativa* potentially affected by selective sweep are color-coded: those in the whole population with red bars, those only in *indica* populations with yellow bars, and those only in *japonica* populations with green bars. The SNP Viewer also graphically shows regions with higher mutation rates (i.e., highly differentiated loci). The genomic coordinates show regions or loci that appear to have been exposed to particular selective pressures through the process of domestication. In addition to RAP-DB (Sakai et al. 2013) and MSU gene models (Ouyang et al. 2007), shown near the top of the viewer, the SNP Viewer now includes Committee on Gene Symbolization, Nomenclature and Linkage (CGSNL) gene names (McCouch 2008) and can instantly supply biological information. For example, Fig. 2 shows the region around Os05g0187500 encoding *GW5* (Shomura et al. 2008), which quantitatively controls rice grain width, and indicates that selective pressure might have been applied in this region.

### SNP Effect Table Function

**Fig. 3:**
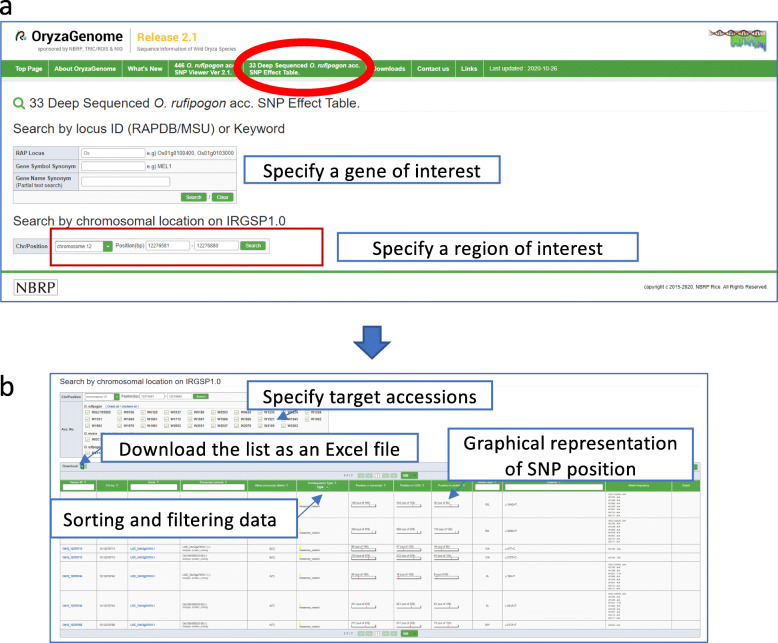
SNP Effect Table. **a** A new function allows users to search for SNPs with specific characteristics in the 33 Deep-Sequenced *Oryza rufipogon* Accessions by gene locus ID or IRGSP1.0 chromosome coordinate, and (**b**) the resultant SNP list is displayed in tabular format

A key update in release 2.1 is a fully customized SNP Effect Table function (Fig. 3), which is designed to search for and identify polymorphisms between the 33 deep-sequenced *O. rufipogon* genotypes and IRGSP1.0 that have specific characteristics. Variant information for 33 *O. rufipogon* accessions (average coverage 19.5×) was obtained by using the SnpEff tool (Cingolani et al. 2012). The SNP Effect Table function provides an output table containing polymorphism information such as position (IRGSP1.0 chromosome coordinate), nearest gene ID, position relative to the nearest gene, type of polymorphism, effect of polymorphism, accessions carrying the polymorphic allele, and so on. SNPs of interest can be searched by gene locus ID, gene symbol synonym, gene name synonym (partial match), or chromosome coordinate (Fig. 3a), and the resultant SNP list is displayed in tabular format (Fig. 3b). The SNP Effect Table can be exported as an Microsoft Excel file. In each entry, the SNP variant type is color-coded, and the position of SNPs in the region is shown on simple map graphs (Fig. 3b).

### Misc Downloads

In the Misc Downloads section in the Downloads page, miscellaneous information used in or associated with OryzaGenome2.1 is provided. Files for tabular-formatted genotype, VCF, and Plink-formatted genotype of newly imputed SNPs of the 446 *O. rufipogon* accessions are available from the Misc Downloads section. These genotype files were newly produced for OryzaGenome2.1 and were made by imputation followed by removal of missing data for use in genome-wide association studies (GWAS) (see Materials and Methods). BED-formatted annotations and *F*st values for the 446 *O. rufipogon* accessions published in Huang et al. (2012) and used in the SNP Viewer are also available at Misc Downloads.

In OryzaGenome1, an initial set of imputation-derived variant information on 446 *O. rufipogon* accessions, which contained missing data, was released. This information is still available from the Misc Downloads section of the Downloads page of release 2.1 (use link “Download imputed SNPs of 446 *O. rufipogon* accessions”) as well as information on these 446 accessions including accession number, habitat/country of origin, ecotype, sequence coverage, and deposited IDs of NGS raw data.

## Conclusions

The goal of OryzaGenome is to facilitate comparative and evolutionary genomic analysis of diverse wild *Oryza* accessions. Most of the wild *Oryza* bioresources used for acquisition of genotype data in OryzaGenome are preserved at the National Institute of Genetics in Japan. These materials were collected during the 1950s to 1980s from around the world, and their biomaterials (such as seeds) are available from Oryzabase (https://shigen.nig.ac.jp/rice/oryzabase/) upon request. Thus, OryzaGenome will help to promote the use of wild *Oryza* bioresources in basic research and speed up the identification of critical agricultural-trait-related genes from these bioresources. OryzaGenome aims to promote rice breeding science as well as potential future crop improvement. OryzaGenome2.1 is a significant update, providing a genus-wide comprehensive genomic repository for wild *Oryza* species.

Improved web-based tools (the SNP Effect Table and SNP Viewer) allow visual inspection of SNPs of interest and the evolutionary background of the surrounding regions in *O. rufipogon*. For genes with known function, it is now possible to quickly narrow down SNPs linked to regions that may affect their function. Lists of SNPs in candidate regions can be quickly obtained on the web interface and downloaded as an Excel file.

### Future Directions

OryzaGenome2.1 provides access to a wide variety of *Oryza* genotypes, including newly sequenced genotypes of seven tetraploid *Oryza* species: *O. minuta* (BBCC), *O. punctata* (BBCC), *O. alta* (CCDD), *O. grandiglumis* (CCDD), *O. latifolia* (CCDD), *O. longiglumis* (HHJJ), and *O. ridleyi* (HHJJ). Compared with other comprehensive databases for *Oryza* genomics (Alexandrov et al. 2015; Wing et al. 2007), OryzaGenome2.1 includes substantially more diverse genotype information. These diverse rice genotypes will be key resources not only for straightforward evolutionary studies, but also for polyploid genome biology in *Oryza* (Shenton et al. 2020). Another important feature of OryzaGenome2.1 is that it offers functions to extract polymorphisms between cultivated rice (*O. sativa*) and its wild progenitor (*O. rufipogon*) and to survey the effect of polymorphisms. These functions will contribute to the analysis of domestication processes and the identification of agriculturally important genes from wild progenitors.

We expect that cross-references between databases storing *Oryza* genome information, such as OryzaGenome2.1, OMAP (http://www.omap.org/index.html), Gramene (http://www.gramene.org), Ensembl Plants (https://plants.ensembl.org/index.html), and Rice SNP seek (https://snp-seek.irri.org), will greatly facilitate evolutionary studies of *Oryza* and contribute to agricultural science. We also foresee that installing and integrating genome browsers for resequencing data, such as TASUKE+, in future versions of OryzaGenome will improve the user experience (Kumagai et al. 2019).

## Supplementary Information

**Additional file 1: Table S1**. Accession list of OryzaGenome2.1.

**Additional file 2: Table S2**. Summary of *K*-mer analysis using genotypes of 217 wild *Oryza* accessions.

**Additional file 3: Table S3** Number of homozygous small nucleotide polymorphisms in 33 *O. rufipogon*.

**Additional file 4: Fig. S1.** Analysis of genome diversity among genus *Oryza*. A. A plot of estimated genome size and estimated repeat length among genus *Oryza*. B. A plot of estimated genome size and estimated repeat content among genus *Oryza*.

## Data Availability

The datasets generated and analyzed during this study are available in OryzaGenome2.1 (http://viewer.shigen.info/oryzagenome21detail/index.xhtml). Accession numbers of short read sequence data are listed in Supplemental Table 1 and on the OryzaGenome2.1 Downloads page (http://viewer.shigen.info/oryzagenome21detail/downloads/index.xhtml). Genetic variation data of 33 *O. rufipogon* accessions are deposited to European Variation Archive under accession numbers PRJEB42581 (Project) and ERZ1714350 (Analyses).

## Abbreviations

CGSNL: Committee on Gene Symbolization, Nomenclature and Linkage
DDBJ: DNA Data Bank of Japan
DRA: DDBJ Sequence Read Archive
GWAS: Genome-wide association studies
NGS: Next-generation sequencing
SNP: Single nucleotide polymorphism
VCF: Variant call format
