# Supplementary material for: OryzaGenome2.1: Database of Diverse Genotypes in Wild Oryza Species
Source: Rice (N Y). 2021 Mar 4;14:24. doi: 10.1186/s12284-021-00468-x (PMC7933306; doi:10.1186/s12284-021-00468-x)
Supplement: Supplementary file 4 — Additional file 4: Fig. S1. Analysis of genome diversity among genus Oryza. A. A plot of estimated genome size and estimated repeat length among genus Oryza. B. A plot of estimated genome size and estimated repeat content among genus Oryza. [file 12284_2021_468_MOESM4_ESM.pdf]

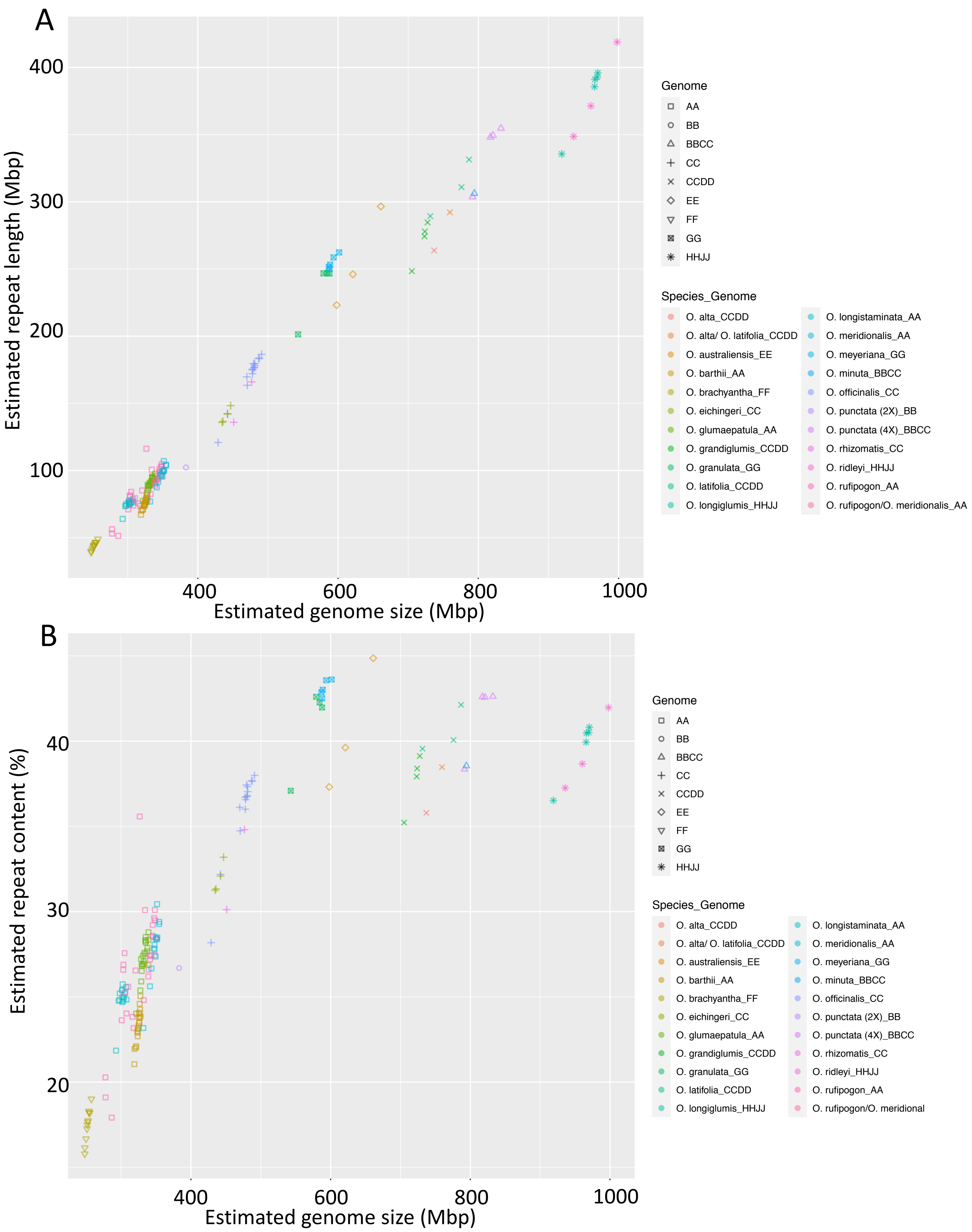

Supplementary Figure 1. Analysis of genome diversity among genus *Oryza*.
A. A plot of estimated genome size and estimated repeat length among genus *Oryza*.
B. A plot of estimated genome size and estimated repeat content among genus *Oryza*.
